# Pathological Clavicular Fracture Following Brachial Plexopathy Occurring Over 50 Years After Radiation Therapy: A Case Report

**DOI:** 10.7759/cureus.82898

**Published:** 2025-04-24

**Authors:** Toshio Netsu, Masato Tsuchiya, Satoshi Kubo, Ryuichi Azuma

**Affiliations:** 1 Department of Plastic and Reconstructive Surgery, National Defense Medical College, Tokorozawa, JPN

**Keywords:** late-onset radiation complications, long-term cancer survivorship, osteoradionecrosis, pathological clavicle fracture, radiation-induced brachial plexopathy, radiation therapy, sarcoma

## Abstract

Radiotherapy is a common cancer treatment that may cause long-term complications such as bone and neurological disorders. This study presents a pathological clavicular fracture that occurred 50 years after radiotherapy for a sarcoma. A 67-year-old female with a history of radiation therapy following surgery at the age of 10 years developed a clavicular fracture without trauma. Prior to the fracture, the patient experienced progressive brachial plexopathy over the past 15 years. Biopsy revealed no malignancy, which led to the diagnosis of a pathological fracture. This study illustrates the delayed onset of radiation-induced complications, with neurological damage appearing earlier than the fracture. Moreover, it underscores the importance of considering radiation-induced bone fragility in patients with unexplained fractures, particularly individuals with prior radiation exposure. Early detection of neurological damage, such as brachial plexopathy, may offer an opportunity for preventive care. Furthermore, it highlights the necessity of patient education regarding the long-term effects of radiation therapy, including bone fragility and the risk of pathological fractures.

## Introduction

Radiotherapy (RT) is an effective cancer treatment that can lead to long-term complications, including neurological and bone disorders [1]. For example, brachial plexopathy is a well-known RT-induced neuropathy following radiation in breast, lymphoma, and head and neck cancers. Following RT, pathological fractures frequently occur in the ribs, pelvis, and femur [2], while clavicular fractures are rare and can occur after radical neck dissection [3]. RT-induced disorders, including brachial plexopathy and pathological bone fractures, are dose-dependent and influenced by the irradiation technique and treatment site.

This study reports a rare case of pathological clavicle fracture following brachial plexopathy occurring more than 50 years after RT for sarcoma, demonstrating the delayed onset of RT-induced complications. It also emphasizes the importance of early detection of neurological damage to educate patients about the potential risk of pathological bone fractures post-treatment.

## Case presentation

A 67-year-old female with a history of sarcoma, treated with radiation following tumor resection at the age of 10 years, was referred to our institution for evaluation and management of a skin ulcer accompanied by a right clavicular fracture. A surviving summary report indicated that the patient received a cumulative dose of 60 Gy during the initial treatment for sarcoma. However, detailed information regarding the radiation technique, including field settings, fractionation, and modality, was unavailable due to the long elapsed time since therapy. Similarly, details of the surgical procedure could not be obtained. The patient reported no recent trauma but described progressively worsening pain in the same area over the past two months. She also noted a gradual decrease in right upper limb muscle strength, which had worsened over approximately 15 years.

**Figure 1:**
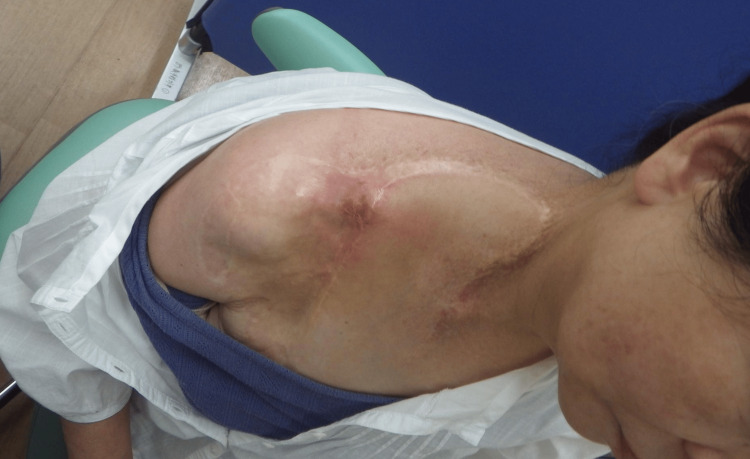
Clinical findings of the first examination. The patient presented with a scar from a previous surgery on her right shoulder, accompanied by a minor skin ulcer.

Upon examination, a small skin ulcer was observed above the clavicle with a palpable clavicular fracture beneath the ulcer (Figure 1). A surgical scar from a deltopectoral flap performed approximately 20 years after RT for the treatment of a radiation-induced skin ulcer was also observed on her right shoulder. Right deltoid muscle atrophy was evident. A manual muscle test revealed a strength score of one in the right biceps brachii, triceps brachii, and brachioradialis and shoulder joint internal rotation, indicating severe muscle weakness. Tendon reflexes of biceps brachii, brachioradialis, and triceps brachii were absent. Sensory function in the right upper arm and ulnar aspect of the forearm diminished.

**Figure 2:**
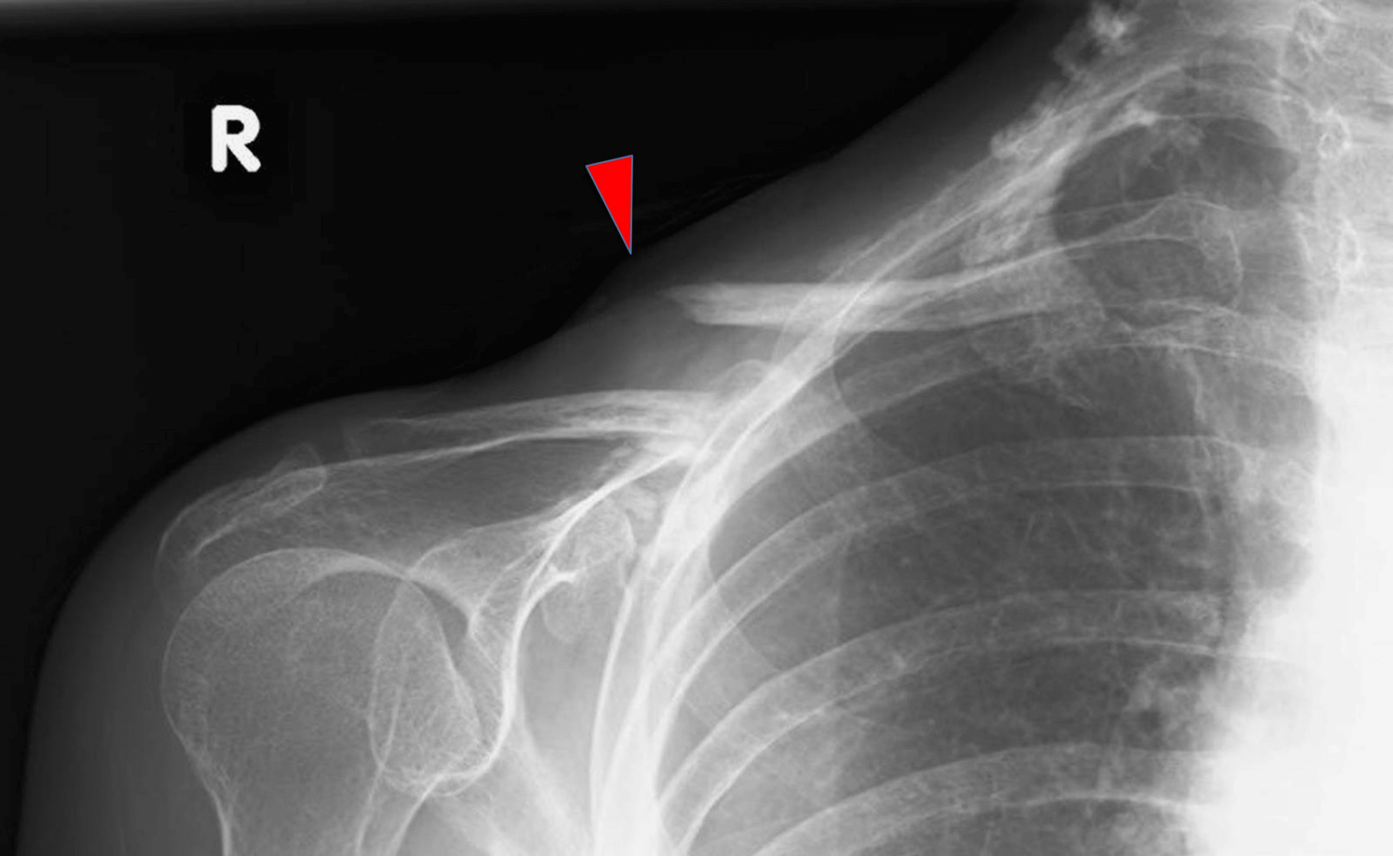
Radiograph of the right shoulder. Arrow indicates the site of the fracture.

**Figure 3:**
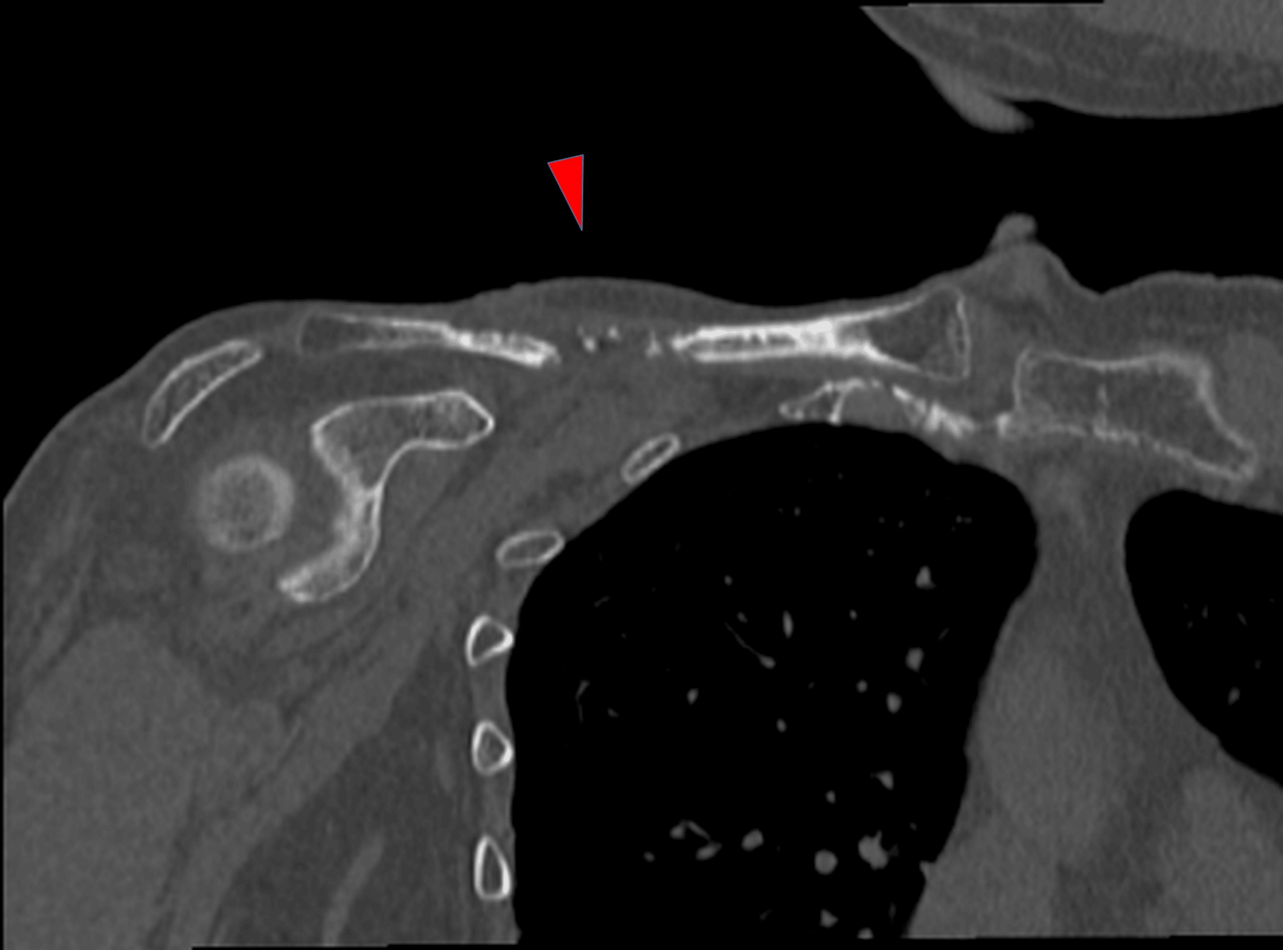
Coronal CT image of the upper limb. Clavicular fracture demonstrated without evidence of osteolytic changes (arrow).

**Figure 4:**
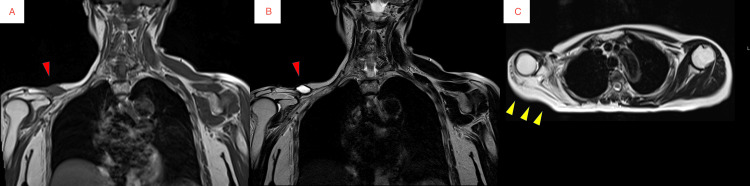
MRI of the right shoulder. (A) Coronal T1-weighted image shows high signal intensity in the right clavicle diaphysis (red arrow). (B) Coronal fat-suppressed T2-weighted image shows corresponding low signal intensity, consistent with bone marrow edema (red arrow). (C) Axial T2-weighted image demonstrates marked atrophy of the deltoid, supraspinatus, and infraspinatus muscles (yellow arrows).

Radiography of the right shoulder revealed a clavicular diaphysis fracture (Figure 2). A CT scan of the upper limb also demonstrated a clavicular fracture without evidence of osteolytic changes (Figure 3). The MRI of the upper limbs revealed bone marrow edema of the clavicle and severe atrophy of the right deltoid, supraspinatus, and infraspinatus muscles (Figures 4A-4C).

A nerve conduction test revealed decreased compound muscle action potential (CMAP) and sensory nerve action potential, along with prolonged latency in the ulnar nerve and decreased CMAP in the median nerve. These findings were consistent with brachial plexopathy and peripheral nerve damage resulting from prior RT.

**Figure 5:**
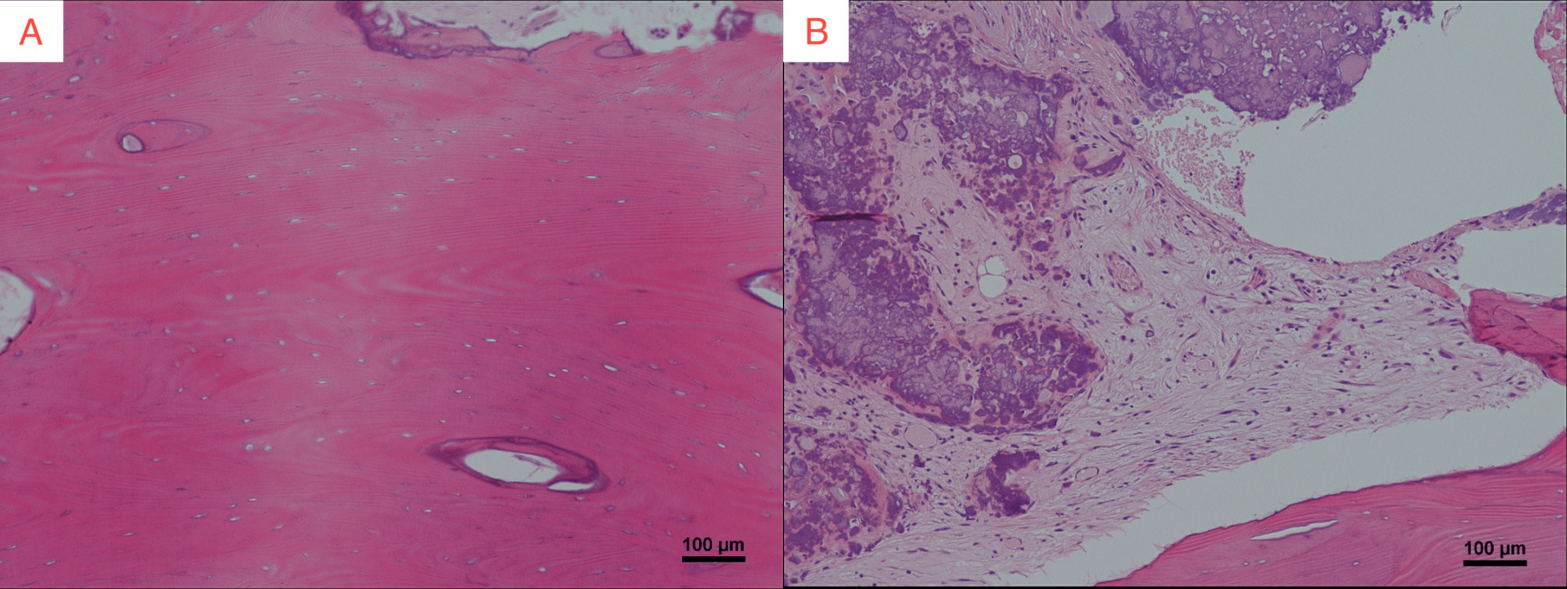
Pathological images of the clavicle biopsy (hematoxylin and eosin staining). (A) Cortical bone (×100): loss of osteocytes within the cortical bone. (B) Trabecular bone (×100): mucinous and fibrous changes, along with loss of fat cells and hematopoietic elements.

Given the patient’s clinical history and the presence of both neurological and skeletal manifestations, a biopsy of the clavicle was performed to further evaluate the lesion, including the possibility of secondary cancer. A biopsy confirmed bone necrosis with no signs of malignancy (Figures 5A, 5B).

**Figure 6:**
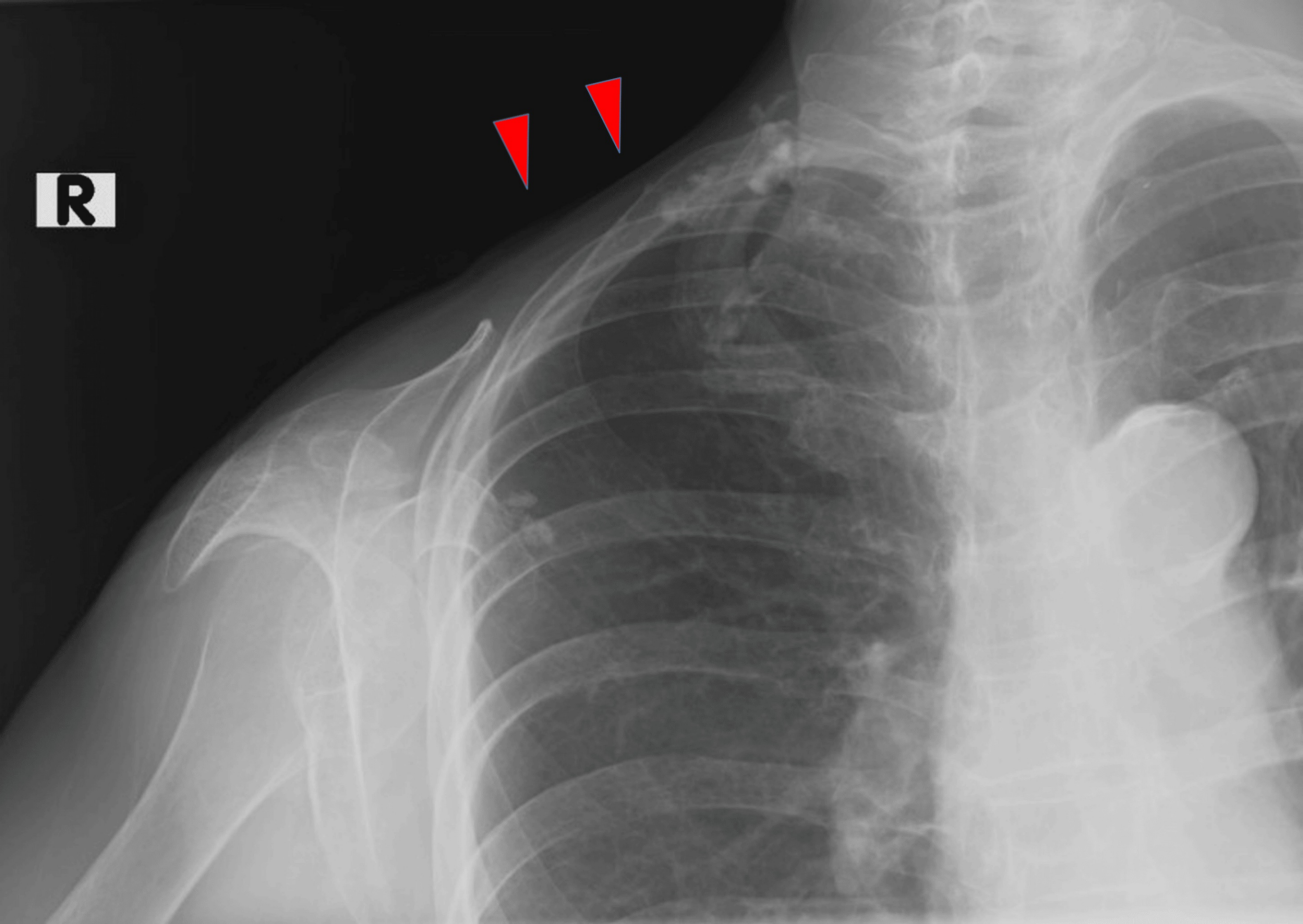
Radiograph of the right shoulder four months after debridement. The arrow points to the area of the clavicle where surgical debridement was performed.

The patient was ultimately diagnosed with a pathological clavicular fracture due to osteoradionecrosis and brachial plexopathy, both of which are late-onset complications of RT. The primary focus of treatment was addressing the skin ulcers that developed as a result of compression from the fractured clavicle. Debridement was performed, and the ulcer was managed conservatively. Follow-up radiographs taken four months after debridement demonstrated satisfactory fracture healing, and there was no recurrence of the ulcer or pathological fracture during the one-year follow-up period (Figure 6).

## Discussion

This study presents a rare pathological clavicle fracture occurring more than 50 years after RT, preceded by chronic neurological symptoms and radiation-related skin complications. Pathological fractures due to RT have been reported with a frequency ranging from 1.2% to 25%, and most of these fractures involve weight-bearing bones, such as the femur, pelvis, and spine [4]. However, a pathological fracture of the clavicle solely caused by RT is a rare complication. Historically, most reported clavicle fractures post-cancer treatment occurred in patients who underwent both neck dissection and post-operative RT, with an incidence of only 0.4-0.5% after neck dissection [5]. The combination of neck dissection and RT can compromise blood supply and structural integrity of the bone, predisposing the clavicle to fracture [5]. However, fractures solely attributable to RT (without head dissection) were reported by Niikura et al. [6]. The latency period for radiation-induced pathological fractures in various cases ranges from 0.4 to 25 years after RT. The median latency in some cases is approximately five to seven years, depending on the type of cancer treated and the extent of irradiation [7].

Generally, the radiation tolerance of the brachial plexus is 60 Gy [8]. The incidence of brachial plexopathy is approximately 5% with radiation doses of 60 Gy or less, but increases exponentially when the dose exceeds 60 Gy. The reported median time from radiation exposure to the onset of paralysis is seven months (range: 4.5-88 months) [9]. In the present case, both brachial plexopathy and pathological clavicular fractures occurred much later than typical reports.

The incidence of RT-induced brachial plexopathy varies in accordance with the irradiation technique, ranging from 66% with historical doses of 60 Gy in 5 Gy fractions to less than 1% with modern doses of 50 Gy in 2 Gy fractions [7]. Several RT-related factors associated with brachial plexopathy have been identified, such as a large total dose (>50 Gy to peripheral nerves and >60 Gy to cranial nerves) and a large dose per fraction (>2.5 Gy). In this case, because the patient had received RT more than 50 years ago, it is likely that the irradiation technique used at that time posed a greater risk. Although modern RT techniques, such as intensity-modulated radiation therapy, have considerably reduced the risk of late adverse effects, this case emphasizes that individuals treated with earlier-generation modalities remain susceptible to delayed complications [10,11]. Therefore, early intervention and prevention, including education, should be prioritized during follow-up.

The patient developed a chronic RT-induced skin ulcer approximately 20 years after RT, which required reconstruction with a deltopectoral flap. Skin ulcers are among the more common late complications of RT and typically occur at lower doses [12,13]. Although this complication preceded the onset of brachial plexopathy and pathological clavicular fracture by a few decades, this case suggests that the presence of superficial complications alone may not reliably predict the development of more severe neurological or skeletal complications, which tend to emerge at higher doses and occur less frequently.

For pathological bone fractures caused by RT, the standard treatment is often a combination of conservative management and surgical intervention. Surgical treatments such as open reduction and internal fixation or bone grafting have been suggested for pathological fractures following post-operative RT [14]. In this case, we decided against performing surgical fixation of the clavicular fracture because of the patient’s pre-existing brachial plexopathy, which caused significant weakness in the deltoid, biceps brachii, triceps brachii, brachioradialis, and shoulder internal rotators. The clavicle functions as a strut that supports shoulder mobility by maintaining the alignment and spacing of the scapula, thereby enabling efficient upper-limb movements. However, because the primary limitation was neural rather than mechanical, fixation is unlikely to improve shoulder joint movements. Therefore, conservative management was considered appropriate.

## Conclusions

This case of pathological clavicle fracture occurring 50 years after RT underscores the importance of recognizing late-onset RT-induced complications. As the brachial plexus and bones have a similar tolerance to RT, clinicians should consider radiation-induced bone damage in patients with a history of RT, particularly when evaluating unexplained fractures. This case also highlights the need for long-term multidisciplinary monitoring of cancer survivors, especially those treated with historical high-dose radiation protocols. Regular follow-ups and patient education are essential for managing these long-term risks.
